# Identifying risk factors for one-year mortality after surgical treatment of periprosthetic femoral fractures around hip arthroplasty

**DOI:** 10.1016/j.jcot.2025.103012

**Published:** 2025-04-08

**Authors:** Maud A.M. Vesseur, Eva H.M. van Beurden, Lee H. Bouwman, Martijn G.M. Schotanus, Raoul van Vugt, Bert Boonen

**Affiliations:** a Department of Orthopedic Surgery, Zuyderland Medical Center, Sittard-Geleen, the Netherlands; b Multidisciplinary Trauma Unit, Zuyderland Medical Center, Heerlen, the Netherlands; c Faculty of Science and Engineering, Maastricht University, Maastricht, the Netherlands; d Maastricht University, Faculty of Health, Medicine and Life Science, Maastricht, the Netherlands; e Department of Surgery, Zuyderland Medical Center, Heerlen, the Netherlands; f School of Care and Public Health Research Institute, Faculty of Health, Medicine and Life Science, Maastricht University, Maastricht, the Netherlands

**Keywords:** Periprosthetic fractures, Reoperation, Mortality, Retrospective studies, Arthroplasty, Replacement, Hip

## Abstract

**Background:**

The increasing number of hip arthroplasties (HA) worldwide, including hemi- and total hip arthroplasties, has led to a rise in periprosthetic femoral fractures (PPFF). These fractures are technically complex, linked to high morbidity and mortality, and impose a burden on healthcare systems. However, clinical guidelines for managing PPFF are lacking, and data on factors affecting postoperative outcomes remain limited. This study aimed to identify risk factors for one-year mortality in surgically treated PPFF patients.

**Methods:**

A retrospective single-center cohort study was conducted on patients with surgically treated PPFF. Data on patient-, implant-, fracture-, surgical- and perioperative characteristics were collected. Univariate and multivariable logistic regression analyses were used to identify independent predictors of one-year mortality.

**Results:**

Among 157 patients (median age was 83 years, 59.2 % ASA III), the one-year overall mortality rate was 16.6 %. Multivariable analyses suggested that residency in a nursing home [OR 4.1 (95 %CI 1.20–14.25) p = 0.025], use of a walking aid [OR 3.6 (95 %CI 1.07–12.36) p = 0.039], initial uncemented stem fixation [OR 4.08 (95 %CI 1.25–13.35) p = 0.020], preoperative urinary bladder catheter (UBC) [OR 4.5 (95 %CI 1.33–15.25) p = 0.016] and lower Body Mass Index (BMI) (Kg/m^2^) [OR 0.87 (95 %CI 0.75–1.00) p = 0.049) were independently associated with one-year mortality.

**Conclusion:**

PPFF patients are frail and highly comorbid. Risk factors such as low BMI, nursing home residency, walking aid use, uncemented stem fixation, and preoperative UBC highlight the need for targeted strategies to improve outcomes.

## 1 Introduction

Hip arthroplasties (HA), first performed in 1891, are among the most common and successful orthopedic procedures worldwide.^1^^,^^2^ The number of HA, including hemi-arthroplasty and total hip arthroplasty, has risen significantly, with data from the UK, Sweden, and New Zealand showing a 37 % increase between 2008 and 2017.^2^^,^^3^ This trend is likely similar in other developed nations and is attributed to aging populations, increasing demand for improved quality of life, and expanded indications for younger, more active patients.^4^^,^^5^ However, the growing number of HA has led to a parallel rise in revision surgeries and traumatic events increasing the incidence of periprosthetic femoral fractures (PPFF).^2^^,^^4^^,^^6^

PPFF around HA represent the most common type of PPFF. The reported risk of a PPFF following HA is about 0.4–3.5 %.^2^^,^^5^ The incidence of PPFF is 3–18 % in HA with uncemented stems and 0.1–1 % in cemented stems.^6^, ^7^, ^8^, ^9^, ^10^, ^11^ PPFF are challenging complications and are associated with high morbidity and mortality rates.^2^^,^^6^ They can occur either intra-or postoperative. The surgical management of PPFF is known to be complex due to the altered anatomy, poor bone quality, and the need to manage both fracture and implant stability.^12^ They often occur in older patients with medical comorbidities and require technically complex operations, resulting in a prolonged hospital stay and increased use of hospital resources.^12^^,^^13^ The associated costs and necessary care are high, placing a significant burden on hospital systems.^13^

Currently there are no established guidelines for the perioperative management of PPFF, and while the Vancouver classification system is useful for classification, it neglects patient- and surgery-related factors that impact outcomes.^14^^,^^15^ In previous research older age, higher American society of Anaesthesiologists classification system (ASA) scores, and delayed surgeries have been independently suggested to increase one-year mortality rates after PPFF.^12^^,^^16^^,^^17^ However, the primary object of these studies was not to identify potential risk factors and therefore a lack in knowledge remains. Due to the high morbidity and mortality associated with PPFF, it is essential to consider these factors in the perioperative management. Limited data regarding patient- and surgical related factors with their influence on the perioperative management of PPFF is available. Therefore, it is needed to get insight into this population to optimize the hospital treatment care path and to ensure patient safety. The first aim of this study was to provide an insight into the patient population suffering PPFF needing surgical management. Second aim was to identify potential risk factors in the perioperative management that have a significant influence on the one-year mortality rate.

## 2 Methods

**Fig. 1:**
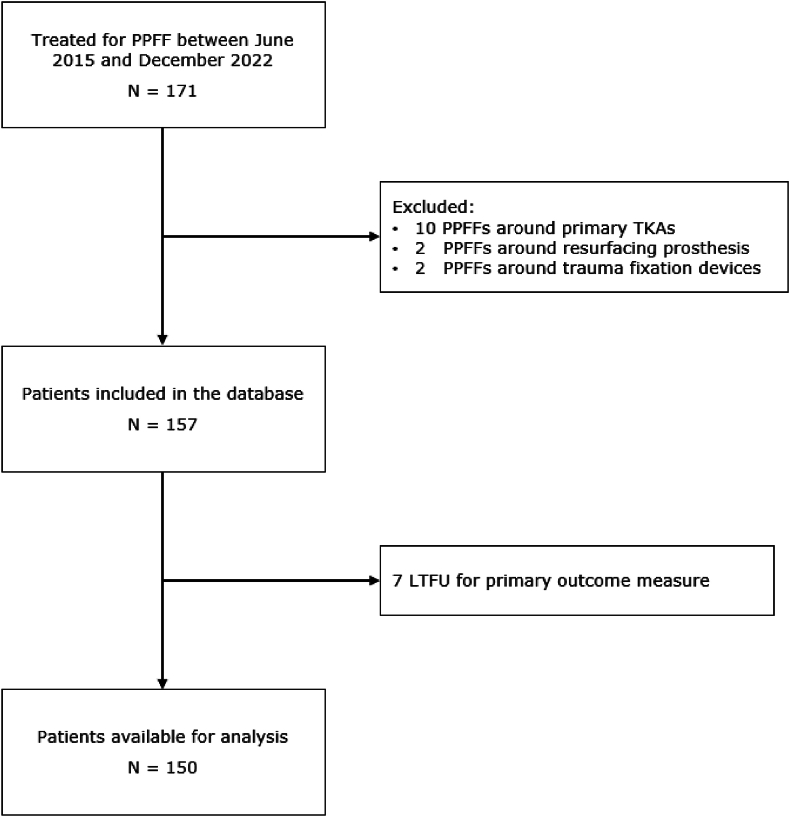
Flowchart PPFF data LTFU, lost to follow up; TKA, total knee arthroplasty.

A retrospective single-center cohort study was conducted investigating all patients who underwent surgical treatment in case of a PPFF around HA between June 2015 and December 2022. Center specific codes for the surgical treatment of PPFF around HA were used extracting our patients of interest. Patients with primary knee arthroplasties, resurfacing prosthesis, PPFF around primary trauma fixation devices such as plates, nails and screws and patients who initially underwent conservative treatment were excluded (Fig. 1).

Data were abstracted from electronic medical records, including information on patient demographics, initial surgery, fracture characteristics, revision surgery, perioperative management, treatment complications (postoperative meaning within two weeks after surgery and after 90 days) and mortality (at 90-days and one-year). An overview of all variables included in the database is given in appendix 1. The primary outcome was defined as one-year mortality after surgical treatment for PPFF around HA.

Fractures were classified by the operating surgeons using the Vancouver Classification system.^18^ The initial stem implants were categorized according to their mode of fixation (cemented or uncemented). Patients of which primary outcome measures were available were included in the analyses for one-year mortality. Due to the large variety, complications were categorized into groups. Patients could have multiple complications. Due to the small numbers, ASA-score I (n = 4) and II (n = 50) were grouped together in the multivariable analyses. For the same reason the ‘wheelchair bound’ subgroup (n = 3) and the weight bearing protocol subgroup ‘other’ (n = 11) were excluded from the multivariable analyses.

Statistical analyses were performed using SPSS software (IBM Corp. Released 2021. IBM SPSS Statistics for Mac, Version 29.0. Armonk, NY, USA: IBM corp). Equal distribution was tested using Kolmogorov-Smirnov analyses. Descriptive statistics were used to describe the total patient population (n = 157). Categorical variables were reported as numbers (n) and percentages (%). Continuous variables were, if normally distributed, reported as mean with standard deviation (±SD and range), or as median with interquartile ranges (IQR) if non-normally distributed. Univariate analyses were performed to compare variables in survivors and non-survivors (n = 150). Patients who were loss to follow up (n = 7) were not included in the univariate analyses. Statistical analysis consisted of an Independent-Samples Median Test for continuous variables and a Chi-square test (X^2^) for categorical variables. Independent variables that showed potential association with one-year mortality in the univariate analyses (defined as P < 0.10) where thereafter used to develop a multivariable logistic regression model to assess their correlation with the dependent variable, one-year mortality after surgical treatment. The multivariable analysis was constructed with binary logistic regression, using two blocks; the first one for pre-operative variables and the second one for post-operative variables. The ‘Backward: Conditional’ method was used to run the analyses, with variables retained based on a backward stepwise approach (conditional P < 0.1). Missing data was coded in SPSS and not used in the analysis for the primary outcome measure. Statistical significance in the multivariable analyses was defined as p < 0.05.

This study was approved by the institutional review board, with code of approval METCZ20230085. It was performed in compliance with the 1975 Declaration of Helsinki, as revised in 2013 and conducted in accordance with the guidelines for Good Clinical Practice.

## 3 Results

### 3.1 Demographics

#### 3.1.1 Patient demographics

**Fig. 2:**
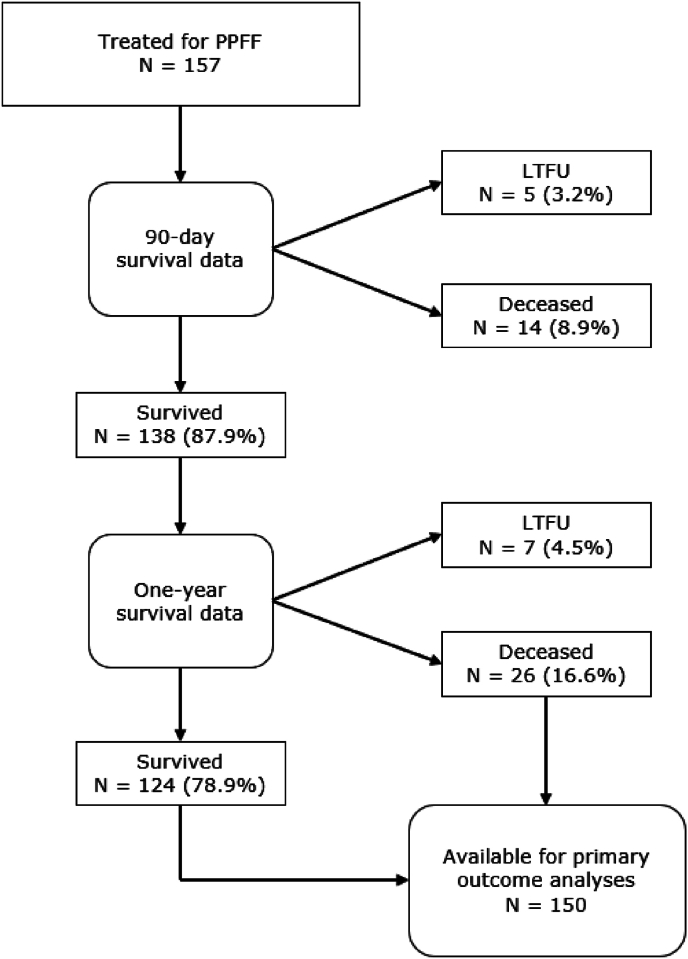
90-day and one-year survival PPFF, periprosthetic femoral fracture; LTFU, lost to follow up.

Out of 171 patients surgically treated for PPFF after HA, a total of 157 patients were considered eligible to participate in our study (Fig. 1). The majority of the population was female (62.4 %), the median age was 83 years (SD ± 12, range 51–95), and the median Body Mass Index (BMI) was 25.6 kg/m^2^ (SD ± 5.4, range 18–41.5). A total of 26 patients (16.6 %) died within one year after surgery for PPFF around HA (Fig. 2). A complete overview of patient demographics is presented in Table 1. Univariate analyses showed that compared to survivors, non-survivors were older (p = 0.049), scored higher on the ASA-classification (p = 0.046), had pre-existing mobility disorders (p = 0.01), and were more likely to suffer from dementia (p = 0.01).

**Table 1:** Patient demographics.

	Value N (%)	Survival N (% within category)	Deceased N (% within category)	P-value
Total no. of patients	157			

Sex				0.227
Male	59 (37.6)	49 (87.5)	7 (12.5)	
Female	98 (62.4)	75 (79.8)	19 (20.2)	
a	0 (0.0)			

Age	83.0 ± 12, 51 - 95	81.0 ± 11.0, 51 - 95	87 ± 7, 61 - 93	0.049∗
a	0 (0.0)			

BMI	25.6 ± 5.4, 18–41.5	26.0 ± 5.1, 18.0–41.5	23.1 ± 5.9, 18.6–33.7	0.018∗
Underweight	2 (1.3)	2 (100)	0 (0.0)	
Healthy weight	69 (43.9)	47 (72.3)	18 (27.7)	
Overweight	61 (38.9)	54 (93.1)	4 (16.0)	
Obese	25 (15.9)	21 (84.0)	4 (16.0)	
a	0 (0.0)			

Place of residence				0.003∗
Own home	113 (72.0)	97 (88.2)	13 (11.8)	
Nursing home	25 (15.9)	15 (60.0)	10 (40.0)	
Residential home	11 (7.0)	8 (88.9)	1 (11.1)	
a	8 (5.1)			

ASA classification				0.046∗
ASA I	4 (2.5)	4 (100)	0 (0.0)	
ASA II	50 (31.8)	45 (93.8)	3 (6.3)	
ASA III	93 (59.2)	67 (76.1)	21 (23.9)	
ASA IV	9 (5.7)	8 (88.9)	1 (11.1)	
a	1 (0.6)			

Preexistent mobility status				0.010∗
Independent	77 (49.9)	68 (93.2)	5 (6.8)	
Walking aid	59 (37.6)	41 (71.9)	16 (28.1)	
Use of a stick	13 (8.3)	9 (75.0)	3 (25.0)	
Wheelchair bound	3 (1.9)	2 (66.7)	1 (33.3)	
a	5 (3.2)			

Pre-existent dementia	22 (14.0)	14 (63.6)	8 (36.4)	0.011∗
a	0 (0.0)			

Osteoporosis	29 (18.5)	24 (85.7)	4 (14.3)	0.637
a	1 (0.6)			

Initial diagnosis				0.742
Coxarthrosis	96 (61.1)	80 (83.3)	16 (16.7)	
Femoral neck fracture	33 (21.0)	26 (83.9)	5 (16.1)	
Other^^^	3 (1.9)	3 (100)	0 (0.0)	
a	25 (15.9)			

BMI, Body Mass Index; ASA, American Society of Anaesthesiologists.

∗ Statistically significant value of p < 0.10.

^Avascular necrosis after intramedullary osteosynthesis (2), epiphysiolysis.

a Missing data.

#### 3.1.2 Fracture and implant characteristics

Table 2 shows an overview of characteristics of the PPFF in the population. The majority of the population suffered from a PPFF caused by low-energy trauma (92.4 %). Most patients sustained a PPFF around total hip arthroplasty (THA) (85.4 %) and the initial method of fixation being uncemented (58.6 %). Fractures classified as Vancouver type B2 were most common in this population (47.8 %). In the univariate analyses, method of stem fixation showed a potential association with one-year mortality (p = 0.087).

**Table 2:** Fracture characteristics.

	Value N (%)	Survival N (%within category)	Deceased N (% within category)	P-value
Initial intervention				0.767
THA	135 (86.0)	107 (82.3)	23 (17.7)	
HA	22 (14.0)	17 (85.0)	3 (15.0)	
				
Initial stem fixation				0.087∗
Cemented stem	65 (41.4)	56 (88.9)	7 (11.1)	
Uncemented stem	92 (58.6)	68 (78.2)	19 (21.8)	
a	0 (0.0)			

Time till fracture (years)	8 ± 11.0, 0 - 29	8 ± 11, 0 - 29	10 ± 13, 0 - 29	0.484
a	23 (14.6)			


Mechanism of trauma				0.777
Low- energy	145 (92.4)	113 (81.9)	25 (18.1)	
High- energy	2 (1.3)	2 (100)	0 (0.0)	
Intra- operative	6 (3.8)	5 (83.3)	1 (16.7)	
Spontaneous	3 (1.9)	3 (100)	0 (0.0)	
a	1 (0.6)			

Fracture side				0.572
Right	83 (52.9)	64 (81.0)	15 (19.0)	
Left	74 (47.1)	60 (84.5)	11 (15.5)	
a	0 (0.0)			

Vancouver classification				0.278
Type A	4 (2.5)	4 (100)	0 (0.0)	
Type B1	32 (20.4)	29 (93.5)	2 (6.5)	
Type B2	75 (47.8)	58 (79.5)	15 (20.5)	
Type B3	11 (7.0)	7 (70.0)	3 (30.0)	
Type C	35 (22.3)	26 (81.3)	6 (18.8)	
a	0 (0.0)			

THA, total hip arthroplasty; HA, hemiarthroplasty.

∗ Statistically significant value of p < 0.10.

a Missing data.

#### 3.1.3 Fracture and implant management

An overview of PPFF management in given in Table 3. The median time of surgery was 107.4 min (IQR 46.5). Most of the patients were treated with open reduction and internal fixation (ORIF) (60.5 %). 64 % (n = 18) of the patients with a Vancouver type B2 fracture treated with ORIF had a cemented HA. Stem revision was done in 62 patients (39.5 %) of which 57 (91.9 %) were treated with uncemented stem fixation. Permissive weight bearing (PWB) was prescribed in the largest proportion after surgery (27.4 %) and full weight bearing was only prescribed in 16 cases (10.2 %). Univariate analyses revealed that patients who were prescribed ‘no weight bearing for six weeks' after surgery had a higher risk for one-year mortality (p = 0.04).

**Table 3:** Fracture management.

	Value N (%)	Survival N (% within category)	Deceased N (%within category)	P-value
Treatment				0.408
Stem revision	2 (1.3)	2 (100)	0 (0.0)	
Stem revision + ORIF	60 (38.2)	46 (78.0)	13 (22.0)	
ORIF	95 (60.5)	76 (85.4)	13 (14.6)	
a	0 (0.0)			

Surgical approach				0.649
Straight lateral	112 (71.3)	87 (82.1)	19 (17.9)	
Anterior	4 (2.5)	4 (100)	0 (0.0)	
Posterolateral	41 (26.1)	33 (82.5)	7 (17.5)	
a	0 (0.0)			

Surgery time (minutes)	107.4 ± 46.5, 43.6–290.0	107.9 ± 46.7, 43.6–290.0	91.2 ± 42.8, 58.1–174.6	0.518
a	0 (0.0)			

Perioperative use of TXA	77 (49.0)	60 (80.0)	15 (20.0)	0.324
a	3 (1.9)			
				
Blood loss	500 ± 500, 100 - 4050	500.0 ± 550.0, 100 - 4050	500 ± 300, 100 - 1700	0.427
a	4 (2.5)			

Method of wound closure				0.714
Staples	100 (63.7)	78 (83.0)	16 (17.0)	
Monocryl intracutaneous	41 (26.1)	32 (80.0)	8 (20.0)	
V-loc intracutaneous	10 (6.4)	9 (90.0)	1 (10.0)	
Vicryl intracutaneous	2 (1.3)	2 (100)	0 (0.0)	
Ethilon transcutaneous	4 (2.5)	3 (75.0)	1 (25.0)	
a	0 (0.0)			

Postoperative weight bearing protocol				
PWB	43 (27.4)	35 (85.4)	6 (14.6)	0.040∗
Full weight bearing	16 (10.2)	11 (68.8)	5 (31.3)	
50 % weight bearing^b^	34 (21.7)	29 (87.9)	4 (12.1)	
No weight bearing^b^	19 (12.1)	9 (52.9)	8 (47.1)	
Plantar contact^b^	34 (21.7)	30 (90.9)	3 (9.1)	
Other^^^	11 (7.0)	10 (100)	0 (0.0)	
a	0 (0.0)			

ORIF, open reduction internal fixation; TXA, tranexamic acid; PWB, permissive weight bearing.

^No weight bearing for two weeks, no weight bearing for eight weeks (2), PWB in combination with hip brace, bed/chair mobilization until full consolidation, 20–30 kg for six weeks (2), no weight bearing until full consolidation, 10 % weight bearing for six weeks, no weight bearing for six weeks with 4 weeks extension brace, 50 % weight bearing for four weeks.

^b^ Statistically significant value of p < 0.10.

a Missing data.

b For a period of six weeks.

#### 3.1.4 In-hospital patient management

Table 4 shows the in-hospital patient management. The median time from hospitalization until surgery was three days (SD ± 3, range 0–14). The median time for hospital discharge after surgery was six days (SD ± 5, range 1–70) and the median clinical follow-up period was five months (SD ± 9, range 2–36). Univariate analyses showed that the independent variables pre-operative urinary bladder catheter (UBC) (p < 0.001), geriatrics consultation (p = 0.016), post-operative UBC (p = 0.032), and blood transfusion (p = 0.038) showed potential of being a predictor for one-year mortality.

**Table 4:** In-hospital patient management.

	Value N (%)	Survival N (% within category)	Deceased N (% within category)	P-value
Time hospitalization till surgery (days)	3.0 ± 3.0, 0 - 14	3.0 ± 4.0, 0 - 13	3.0 ± 3, 1 - 14	0.727
a	0 (0.0)			

Preoperative DOS-score	1.3 ± 4.0, 0 - 11	1.3 ± 3.0, 0 - 10	2.7 ± 5, 0 - 11	0.782
a	92 (58.6)			

Preoperative pain-score	2.0 ± 2.0, 0 - 9	2.0 ± 2.0, 0 - 9	2.0 ± 3, 0 - 7	0.951
a	21 (13.4)			

Preoperative UBC	79 (50.3)	55 (72.4)	21 (27.6)	<0.001∗
a	0 (0.0)			

Geriatrics in consultation	99 (63.1)	74 (77.1)	22 (22.9)	0.016∗
a	0 (0.0)			

Postoperative DOS-score	1.0 ± 4.0, 0 - 9	1.3 ± 3.0, 0 - 10	1.7 ± 4.0, 0 - 9	0.818
a	93 (59.2)			

Postoperative pain score	2.0 ± 3.0, 0 - 7	2.0 ± 3.0, 0 - 7	2.0 ± 3.0, 0 - 7	0.619
a	6 (3.8)			

Postoperative UBC	121 (77.1)	90 (78.9)	24 (21.1)	0.032∗
a	0 (0.0)			

Hospital discharge after surgery (days)	1.0 ± 4.0, 0 - 18	1.0 ± 5.0, 0 - 18	1.0 ± 3.0, 0 - 10	0.693
a	3 (1.9)			

Blood transfusion	93 (59.2)	68 (77.3)	20 (22.7)	0.038∗
a	0 (0.0)			

Days till first mobilization	1.0 ± 1.0, 0 - 5	1.0 ± 1.0, 0 - 5	2.0 ± 1.0, 1 - 4	0.220
a	0. (0.0)			

Complication rate				
Post-op	60 (38.2)	42 (75.0)	14 (25.0)	0.060∗
a	1 (0.6)			
90 days	22 (14.0)	19 (86.4)	3 (13.6)	0.620
a	5 (3.2)			

Hospital discharge after surgery (days)	6.0 ± 5.0, 1 - 70	6.0 ± 5.0, 1 - 70	7.0 ± 2.0, 3 - 17	0.438
a	3 (1.9)			
				
Follow up (months)	5.0 ± 9.0, 2 - 36	5.0 ± 9.0, 2 - 36	4.5 ± 3.0, 3 - 7	0.263
a	73 (46.5)			

DOS, Delerium observation screening; UBC, Urinary Bladder Catheter.

∗ Statistically significant value of p < 0.10.

a Missing data.

#### 3.1.5 Complications

An overview of complications developed postoperative and at 90 days after surgical treatment in given in Table 5. Most of the complications postoperatively were of urogenital nature, accountable for 14.0 %. The complication rate at 90 days postoperatively was mostly defined by dislocations of the hip (7.0 %, n = 11). Univariate analyses showed that patients with post-operative complications had a higher risk for one-year mortality (p = 0.06). Out of the 11 patients with a dislocation within 90-days, mortality was 27.3 % (n = 3).

**Table 5:** Complications postoperative and at 90-days.

Postoperative (Total 83 = 52.87 %)	Group	Value N (% within category, % total group)
	Cardiovascular	21 (25.3, 13.4)
	Pulmonal	7 (8.4, 4.5)
	Urogenital	22 (26.5, 14.0)
	Neurological	5 (6.0, 3.2)
	Gastrointestinal	3 (3.6, 1.9)
	Dermatological	13 (15.7, 8.3)
	Hip dislocation	5 (6.0, 3.2)
	Other^a^	7 (8.4, 4.5)

**90-days (Total 24 = 15.29 %)**		

	Hip dislocation	11 (45.8, 7.0)
	Surgical site infection	8 (33.3, 5.1)
	Dermatological	2 (8.3, 1.3)
	Other^b^	3 (12.5, 1.9)

a COVID (2), bacteremia, electrolyte imbalance (2), bone fissure, insufficient fracture reduction.

b Non-union, cable failure, trochanteric bursitis.

### 3.2 Multivariable analysis

Displayed in Table 6 are the independent variables used to create a multivariable logistic regression model. After accounting for multiple independent variables, analyses showed the odds of mortality to be four times higher for patients with initial uncemented stem fixation compared to cemented stem fixation [4.08 (95 %CI 1.25–13.35)] (p = 0.020). Patients who lived in a nursing home had a four times higher risk of one-year mortality in comparison to patients who lived independently [4.13 (95 %CI 1.20–14.25)] (p = 0.025) and patients that mobilized using a walking aid pre-trauma had a 3.6 times higher risk for one-year mortality compared to patients who mobilized independently [3.63 (95 %CI 1.07–12.36)] p = 0.039). Additionally, patients having a UBC preoperative showed 4.5 times higher odds for mortality in comparison to patients who did not [4.50 (95 %CI 1.33–15.25)] (p = 0.016). An increase of 1 kg/m^2^ in BMI, however, independently correlates with a 13 % decrease in relative odds of mortality [0.87 (95 %CI 0.75–0.99)] (p = 0.049). At the same time, older age, higher ASA-score, post-operative non weight bearing, and pre-existent dementia were shown not to be independent predictors for one-year mortality.

**Table 6:** Predictors of one-year mortality after PPFFs.

	Adjusted Odds Ratio (95 %CI)	P-value
BMI (kg/m^2^)	0.87 (0.75–1.00)	0.049∗

Place of residence		
Own home (reference)		0.075
Nursing home	4.13 (1.20–14.25)	0.025∗
Residential home	1.03 (0.08–13.62)	0.982

Preexistent mobility status		
Independent (reference)		0.081
Walking aid	3.63 (1.07–12.36)	0.039∗
Use of a stick	5.07 (0.70–36.86)	0.109
Preoperative UBC	4.50 (1.33–15.25)	0.016∗
		
Initial stem fixation		
Cemented stem (reference)		
Uncemented stem	4.08 (1.25–13.35)	0.020∗

BMI, Body Mass Index; UBC, Urinary Bladder Catheter.

∗ Statistically significant value of p < 0.05.

## 4 Discussion

This single-center retrospective cohort study aimed to provide insights into the frail patient population requiring surgical treatment for PPFF around HA and to identify risk factors for one-year mortality. The median age was 83 years, and most patients were classified as ASA-score III, indicating severe comorbidities, consistent with previous research.^16^^,^^19^^,^^20^ Independent predictors for one-year mortality included initial uncemented stem fixation, preoperative UBC use, walking aid dependence, nursing home residency, and low BMI (<25.6 kg/m^2^).

This study did not identify older age, higher ASA-score, post-operative non weight bearing, or pre-existent dementia as independent predictors of one-year mortality, contrasting with prior studies suggesting these factors, along with male sex, as significant predictors in PPFF management.^16^^,^^17^^,^^21^, ^22^, ^23^ Similarly, Khassawna et al. also found no association between ASA-score (III/IV vs I/II) and mortality, aligning with our findings.^19^ Surgical delay has been debated as a predictor. Bhattacharyya et al. reported that delays beyond two days significantly increased one-year mortality in PPFFs around THA.^12^ However, Finlayson et al. found no correlation between delays exceeding 48 h and mortality in PPFF around HA, consistent with our results.^16^ The discrepancies in the literature may stem from small sample sizes and low statistical power. This study utilized a robust database and multivariable analysis accounting for data dependencies, which were often unaddressed in prior research. Therefore, our findings offer a reliable perspective on mortality predictors in PPFF management.

In the current study we observed a one-year mortality rate of 16.6 %, slightly higher than in previous reports (10.7 %–13.2 %).^12^^,^^16^^,^^19^^,^^21^^,^^22^ An increase of 1 kg/m^2^ in BMI was associated with a 13 % reduction in relative odds of mortality, aligning with Drew et al., who found a significant link between low BMI (<25 kg/m^2^) and increased mortality.^21^ However, other studies found no correlation, likely due to small sample sizes and unequal BMI subgroup distributions.^20^^,^^22^ By adjusting for factors like age and ASA-score, our analysis provides a clear understanding of BMI's role. Research on elderly hip fracture patients supports our findings, indicating that a BMI of 25.0–29.9 kg/m^2^ significantly reduces one-year mortality risk.^24^

Patients with uncemented stem fixation had a fourfold higher risk for one-year mortality than those with cemented stem fixation. Previous studies, reported no significant correlation between stem fixation method and mortality.^19^^,^^22^ This discrepancy may be due to the higher incidence of PPFF around uncemented stems and differences in surgical management. Vancouver B2 fractures involving uncemented stems often require stem revision due to compromised fixation, whereas fractures around cemented stems can sometimes be treated with ORIF, depending on cement integrity. In this study, stem revision was more commen in uncemented stems (n = 51) than cemented stems (n = 11). Revision procedures, linked to longer operative time, greater blood loss and higher physiological stress, may contribute to increased complications and mortality. Additionally, uncemented stems are typically used in younger, more active patients, but PPFF in this group may indicate poor bone quality, leading to severe fractures and complex surgeries. Conversely, cemented stems, often chosen for older, frailer patients, provide immediate stability and may lower early postoperative risks. While prior research has not consistently found a mortality difference based on fixation type, our findings underscore the need for further investigation into surgical management and patient outcomes.

Patients residing in nursing homes and those who used a walking aid for mobility prior to treatment were at higher risk of one-year mortality. This outcome corresponds to the findings of Nassar et al., who also found a significant difference in these variables in the univariate analyses between survivors and non-survivors.^22^ These results are inconclusive as it is not clear if these variables were accounted for in the multivariable analyses in their study.

Preoperative UBC use was associated with a four and a half fold increase in mortality risk. To our knowledge, no prior research has examined this variable, preventing direct comparison with other studies. Previous studies on UBC use in hip fracture surgery patients has linked indwelling catheters to higher rates of urinary tract infections (UTI) and postoperative urine retention (POUR), which may increase the risk of chronic renal insufficiency and urosepsis.^25^, ^26^, ^27^ these complications could contribute to the elevated mortality observed in patients with preoperative UBC use.

Our study found that one-year mortality was influenced by patient-related factors (preoperative UBC and low BMI) rather than fracture-, implant- or surgery-related factors. While variables like BMI, use of a walking aid, initial uncemented stem fixation and residency in a nursing home by admission are non-modifiable, they help identify high-risk patients requiring closer in-hospital management. Preoperative UBC, however, is modifiable, and intermittent catheterization or early removal of indwelling catheters may reduce UTI and POUR risks, potentially improving outcomes.^25^ However, further research is needed for definitive recommendations.

Univariate analyses identified a ‘no-weight-bearing’ protocol as potential predictor of one-year mortality. Despite adjustment in multivariable analysis, early weight-bearing should be prioritized unless contraindicated. Immobilization increases the risk of complications such as pressure ulcers, UTIs, pneumonia and delirium. Weight-bearing as tolerated promotes bone healing, independence, and shorter hospital stays. Early mobilization is therefore recommended post-surgery.

Furthermore, the present study did not investigate the pre-operative nutritional status of the included patients. Seeing as lower BMI has shown to be an independent predictor of mortality and the population is described to be frail and of older age, it might be interesting for future research on this topic to assess nutritional status, start with protein-enriched diet and see its effect on the rehabilitation process and mortality rates.

The study population, characterized by old age and high ASA-scores, aligns with research on proximal femoral fractures; an extremely vulnerable population.^28^ Prior studies have recommended optimizing in-hospital care, including delirium prevention, early geriatric consultation, minimal surgical delay and rapid mobilization.^29^ Given the similarities, these strategies should also be integrated into PPFF treatment guidelines.

A strength of this study is its large patient population from a high-volume trauma center, making it one of the first to examine mortality predictors in PPFF around HA. However, limitations include potential confounding due to its retrospective design, missing data on in-hospital management, and grouping of complications without assessing severity.

This retrospective study highlights the frailty of PPFF patients after HA, characterized by old age and poor health (ASA-score III). Independent predictors of one-year mortality include low BMI, preoperative UBC, nursing home residency, walking aid use, and uncemented stem fixation. Further research with larger cohorts is needed to refine clinical guidelines and optimize perioperative management.

## 5 Learning points and clinical inferences

• *Patient frailty and risk factors for mortality*
  o This study highlights that patients with PPFF around HA are generally frail, with a median age of 83 years and a high prevalence of comorbidities (ASA-score III).
  o Clinical Inference: Identifying high-risk patients (e.g., those in nursing homes, those who use walking aids, and those with a low BMI) allows for targeted perioperative strategies to improve outcomes.
• *Preoperative UBC and mortality*
  o Patients with preoperative UBC had a four and a half fold higher risk of one-year mortality, potentially due to complications like UTI and sepsis.
  o Clinical Inference: Avoiding prolonged catheter use and implementing early removal strategies can help reduce postoperative complications and improve survival.
• *Weight-bearing and early mobilization*
  o A ‘no-weight-bearing’ postoperative protocol was associated with increased mortality risk. Encouraging early mobilization can reduce complications like pressure ulcers, pneumonia, and delirium.
  o Clinical Inference: Implementing weight-bearing as tolerated or PWB protocols should be a priority in postoperative care to enhance recovery and reduce hospital stay.
• *Nutritional status and BMI as predictors of survival*
  o A lower BMI (<25.6 kg/m^2^) was an independent predictor of increased mortality, with each 1 kg/m^2^ increase reducing mortality risk by 13 %.
  o Clinical Inference: Preoperative nutritional assessment and interventions, such as protein-enriched diets, should be considered to optimize patient resilience and rehabilitation outcomes.
• *Uncemented stem fixation increases mortality risk*
  o Patients with initial uncemented stem fixation had a four times higher risk of one-year mortality compared to cemented stem fixation.
  o Clinical Inference: Surgeons may need to reconsider implant selection, particularly in frail elderly patients, to reduce long-term complications and mortality risks.

## 6 Potential conflicts of interest and funding sources

All authors certify that they have no affiliations with or involvement in any organization or entity with any financial interest or non-financial interest in the subject matter or materials discussed in this manuscript.

## Patient's consent

This study was conducted as a retrospective analysis using anonymized data obtained from existing medical records. No personally identifiable information was collected, stored, or analyzed, ensuring complete confidentiality and compliance with data protection regulations. Due to the retrospective nature of the study and the use of de-identified data, specific patient consent was not required in accordance with the ethical guidelines, e.g., the Declaration of Helsinki and approval by the specific ethics review board from our institution.

## CRediT authorship contribution statement

**Maud A.M. Vesseur:** Conceptualization, Methodology, Software, Validation, Investigation, Formal analysis, Resources, Data curation, Writing – original draft, Visualization, Project administration. **Eva H.M. van Beurden:** Software, Validation, Investigation, Formal analysis, Resources, Data curation, Writing – original draft, Visualization. **Lee H. Bouwman:** Software, Validation, Writing – review & editing, Visualization, Supervision, Project administration. **Martijn G.M. Schotanus:** Conceptualization, Methodology, Software, Validation, Writing – review & editing, Supervision, Project administration. **Raoul van Vugt:** Conceptualization, Methodology, Writing – review & editing, Supervision, Project administration. **Bert Boonen:** Conceptualization, Methodology, Writing – review & editing, Supervision, Project administration.

## Ethical statement

This study was approved by the Zuyderland Medical Center institutional review board, with code of approval METCZ20230085 (April 2023). It was performed in compliance with the 1975 Declaration of Helsinki, as revised in 2013 and conducted in accordance with the guidelines for Good Clinical Practice.

## Funding statemnent

This research did not receive any specific grant from funding agencies in the public, commercial or not-for-profit sectors.

## Declaration of competing interest

The authors declare that they have no known competing financial interests or personal relationships that could have appeared to influence the work reported in this paper.
