# Supplementary material for: Identifying risk factors for one-year mortality after surgical treatment of periprosthetic femoral fractures around hip arthroplasty
Source: J Clin Orthop Trauma. 2025 Apr 8;66:103012. doi: 10.1016/j.jcot.2025.103012 (PMC12237752; doi:10.1016/j.jcot.2025.103012)
Supplement: Multimedia component 1 [file mmc1.docx]

**Appendix**

Appendix 1: Table S1. Overview of variables included in the database.

| **Patient demographics** | **Fracture and implant characteristics** | **Fracture and implant management** | **In-hospital patient management** | **Other** |
| --- | --- | --- | --- | --- |
| Sex | Initial intervention | Revision treatment | Time hospitalization till surgery | Difference discharge location from initial place of residence |
| Age | Date of initial intervention | Revision surgeon | Preoperative DOS-score | Frequent falls before surgery |
| Zipcode | Date of PPFF | Revision surgical assistant | Preoperative pain-score | Recurrence of falls |
| Length | Time initial surgery till fracture | Revision surgical approach | Preoperative UBC | Osteoporosis screening done |
| Weight | Mechanism of trauma | Type of anesthetics used | Geriatrics in consultation | Time till union fracture |
| BMI | Fracture side | Use of regional anesthetics blocks | Dietetics in consultation | Limitations in mobility after surgery |
| Place of residence | Vancouver class | Surgery time | Blood transfusion | Other limitations after surgery |
| ASA | Initial surgical approach | Perioperative use of TXA | Hb decrease | Definite change in place of residence |
| Preexistent mobility status | Initial implant type | Blood loss | Re-transfusion | Date of death |
| Preexistent dementia | Initial surgeon | Use of cell saver | Postoperative Dos-score | Consultations postoperative and reason |
| Osteoporosis yes/no | Initial surgical assistant | Type of implant | Postoperative pain score | Follow-up |
| Initial diagnosis |  | Additional use of osteosynthesis material | Postoperative UBC |  |
| Mortality 90-days |  | Method of wound closure | Number of days wound leakage |  |
| Mortality one-year |  | Postoperative load bearing protocol | Days till first mobilization |  |
|  |  |  | Complications postoperative |  |
|  |  |  | Complications after 90-days |  |
|  |  |  | Hospital discharge after surgery |  |
